# Supplementary material for: A predictive model for left ventricular reverse remodeling after pharmacological therapy in children with recent-onset dilated cardiomyopathy
Source: PLoS One. 2025 Apr 1;20(4):e0321126. doi: 10.1371/journal.pone.0321126 (PMC11960990; doi:10.1371/journal.pone.0321126)
Supplement: S1 File — (DOCX) [file pone.0321126.s001.docx]

**Supplementary tables 1 The collinearity diagnostic analysis of factors for predicting LVRR in children with DCM.**

| **Variable** | **Age** | **SCr** | **BUN** | **HB** | **LVEDD z-score** | **LVEF** | **QRS Interval** |
| --- | --- | --- | --- | --- | --- | --- | --- |
| **Tolerance** | 0.319 | 0.316 | 0.801 | 0.710 | 0.797 | 0.819 | 0.753 |
| **VIF** | 3.133 | 3.168 | 1.248 | 1.409 | 1.254 | 1.221 | 1.328 |

SCr, serum creatinine; BUN, blood urea nitrogen; HB, hemoglobin; LVEDD, left ventricular volume and end-diastolic dimension; LVEF, left ventricular ejection fraction

**Supplementary tables 2 Univariate and multivariate logistic regression analysis of candidate predictors variable after excluding first year death cases**

| **Predictor Variable** | **Univariate analysis**  **OR (95%CI)** | **P-value** | **Multivariate analysis**  **OR (95%CI)** | **P-value** |
| --- | --- | --- | --- | --- |
| **Age (years)** | 0.75 (0.67–0.84) | <0.001 | 0.73 (0.61–0.89) | 0.002 |
| **SCr (µmol/L)** | 0.94 (0.92–0.97) | <0.001 | 1 (0.95–1.04) | 0.903 |
| **BUN (mmol/L)** | 0.81 (0.67–0.98) | 0.03 | 0.9 (0.69–1.18) | 0.457 |
| **HB (g/L)** | 0.96 (0.94–0.98) | 0.001 | 0.97 (0.94–1.01) | 0.12 |
| **LVEDD z-score** | 0.82 (0.68–1) | 0.046 | 0.57 (0.39–0.82) | 0.002 |
| **LVEF (%)** | 1.02 (0.98–1.07) | 0.232 | 0.98 (0.92–1.04) | 0.506 |
| **QRS interval duration (ms)** | 0.91 (0.87–0.95) | <0.001 | 0.92 (0.87–0.97) | 0.01 |

OR, odds ratios; CI, confidence intervals; SCr, serum creatinine; BUN, blood urea nitrogen; HB, hemoglobin; LVEDD, left ventricular volume and end-diastolic dimension; LVEF, left ventricular ejection fraction
