# Supplementary material for: A predictive model for left ventricular reverse remodeling after pharmacological therapy in children with recent-onset dilated cardiomyopathy
Source: PLoS One. 2025 Apr 1;20(4):e0321126. doi: 10.1371/journal.pone.0321126 (PMC11960990; doi:10.1371/journal.pone.0321126)
Supplement: Attachment — Submitted filename: Response to Reviewers.pdf [file pone.0321126.s003.pdf]

Dear Editor and Reviewers,

We sincerely thank you for your thorough review and constructive comments on our manuscript. We have carefully addressed all the points raised and made corresponding revisions to improve our manuscript. Below are our point-by-point responses to each comment.

Response to Reviewer #1:

Comment 1 (Line 84: Heart failure symptoms in pediatric populations)

We greatly appreciate the reviewer's insightful comment regarding the need for more detailed information about age-specific heart failure symptoms. We have made the following comprehensive modifications:

1. **We have enhanced Table 1 by adding detailed heart failure symptoms with age-specific categorization** (marked in red in the manuscript):

1) For children aged 5-18 years:

* Documented chest pain and fatigue
* Notably, fatigue showed significantly higher prevalence in the non-LVRR group (68.1% vs 12.7%, p<0.001)

2) For children under 5 years:

* Documented failure to thrive and feeding difficulties
* Both symptoms were significantly more common in the LVRR group (41.8% and 36.4% respectively, p<0.001)

3) Age-independent symptoms including dyspnea and gastrointestinal complaints have also been included
4) Due to the addition of detailed symptom information, Table 1 became lengthy. Therefore, we have:

* Reorganized Table 1 to focus on clinical characteristics and symptoms；
* Created a new Table 2 for discharge medication data

Comment 2 (Lines 126-129: LVRR definition references)

We thank the reviewer for highlighting the importance of supporting references for

our LVRR definition. We have strengthened this section by:

1. Adding relevant references and explanations in the discussion section （marked in red in the manuscript）：

"The definition of LVRR has evolved over time and has not yet been fully standardized. Early studies primarily focused on the improvement of cardiac function, but as research progressed, the evaluation parameters gradually expanded to include multiple indicators, encompassing changes in left ventricular volume and morphology[23]."

2. Including citations demonstrating "the prognostic value of both functional and structural recovery[7, 24-26]" in pediatric DCM, which supports our comprehensive definition incorporating both functional (LVEF ≥55%) and structural (LVEDD Z-score ≤2) parameters.

Comment 3 (Line 172: Mortality causes)

We appreciate this important question regarding the 58 deaths in our study. We chose all-cause mortality as our endpoint for several methodologically sound reasons:

1. Population characteristics:

1) Our cohort consisted exclusively of pediatric patients with primary DCM
2) These patients typically lack adult-type comorbidities (e.g., diabetes, hypertension)

2. Mortality patterns:

1) Cardiovascular events were the predominant cause of death
2) Even in non-cardiac deaths, underlying DCM likely contributed to outcomes
3) Methodological considerations:
4) This approach maintains scientific rigor
5) Avoids potential bias in cause-of-death classification
6) Aligns with established landmark studies in pediatric DCM

Comment 4 (Lines 247-249: Model accuracy by age group)

We thank the reviewer for this important question about age-specific model performance. We have conducted detailed analyses across three age groups:

1. Model validation results:

1) 1-10 years (n=64): AUC 0.974 (95% CI: 0.918~1.000)

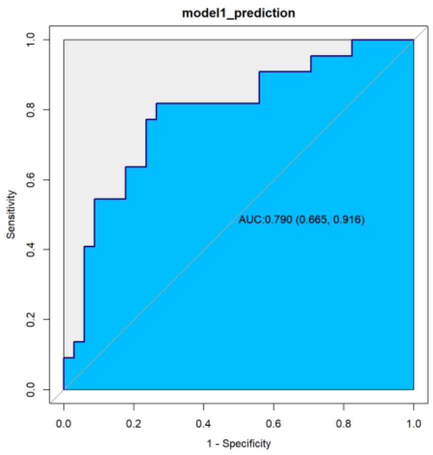

2) >10 years (n=52): AUC 0.904 (95% CI: 0.802~1.000)

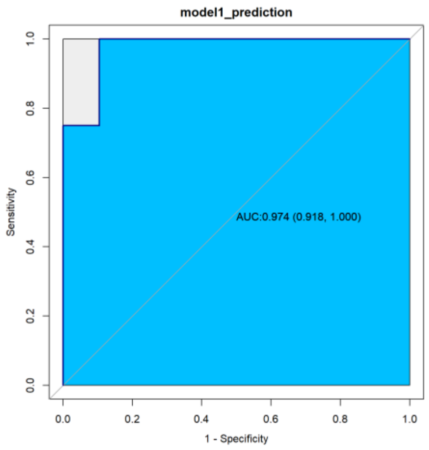

3) <1 year (n=30): AUC 0.790 (95% CI: 0.665~0.916)

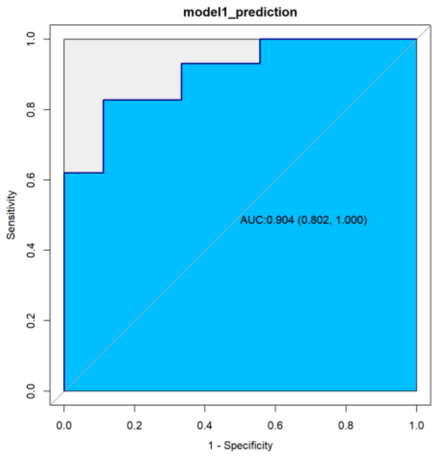

2. Key findings:

1) Consistent good performance across age groups
2) Most stable prediction in the 1~10 years group
3) Wider confidence intervals in smaller subgroups reflect sample size limitations

Comment 5 (Figure 1: Phrasing)

We appreciate the reviewer's attention to detail. We have revised the first box to read:

"224 children younger than 18 years hospitalized with DCM between January 2012 and June 2023"

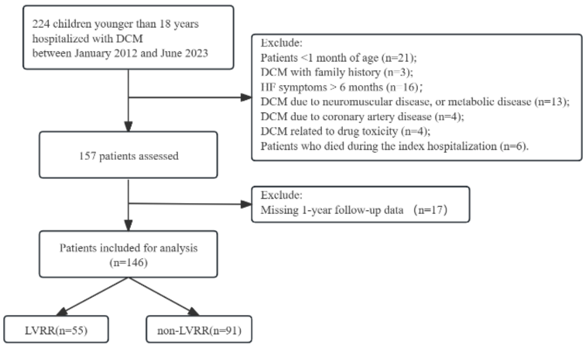

Response to Reviewer #2:

Comment 1 (External validity)

We appreciate this constructive feedback regarding external validity limitations. We have expanded our discussion to address:

1) Implications of single-center, retrospective design
2) Potential selection bias effects
3) Impact of center-specific factors
4) Need for multi-center prospective validation studies
5) Rewritten this limitation in the Discussion section (marked in red in the manuscript)

Comment 2 (Genetic and ethnic variations)

We thank the reviewer for highlighting this important aspect. We have:

1) Acknowledged the potential influence of genetic and ethnic factors on LVRR

2) Added this limitation to our discussion
3) Emphasized the need for future studies addressing these factors
4) Rewritten this limitation in the Discussion section (marked in red in the manuscript)

Comment 3 (Follow-up duration variability)

We appreciate this methodological observation and have addressed it through multiple approaches. We have rewritten this limitation in the Discussion section (marked in red in the manuscript):

1. Statistical considerations:

1) Conducted sensitivity analysis excluding early mortality cases
2) Implemented time-to-event analysis with appropriate censoring
3) Added detailed methodology explanations

2. Clinical context:

1) Noted that LVRR typically occurs within 12-18 months post-diagnosis
2) Confirmed our minimum follow-up (15 months) exceeds this window

Comment 4 (Comparison with adult predictive models)

We greatly appreciate your suggestion regarding the comparison with adult predictive models. After a thorough review of the adult LVRR literature, we have incorporated a comprehensive comparison in the Introduction section (marked in red in the manuscript):

Key Predictors:

1) Adult studies often include factors such as hypertension history and symptom duration, which may not apply to pediatric populations.
2) Importantly, both adult and pediatric models consider LVEDD and LVEF as crucial predictors of LVRR.

Novelty in Pediatric Context:

1) Our model uniquely focuses on age-specific predictors relevant to children, such as developmental status and the absence of adult-related comorbidities.
2) By excluding predictors like hypertension, our model reflects the distinct pathophysiology of pediatric DCM.

Clinical Implications:

1) While adult models provide valuable insights, pediatric DCM requires tailored predictors and scoring systems to accurately reflect the unique characteristics of the population.
2) Our findings may enhance the risk stratification methods available for clinicians treating pediatric patients with DCM.
3) We have further expanded the discussion section to provide this comparison and explain the rationale behind our chosen predictors, emphasizing the difference between adult and pediatric predictive models.

We believe these revisions have substantially strengthened our manuscript and addressed all concerns raised by both reviewers. We are grateful for their valuable input that has helped improve the quality and clarity of our work.

Sincerely,

Pang,
